# Fe–Pd nanoflakes decorated on leached graphite disks for both methanol and formic acid electrooxidation with excellent electrocatalytic performance

**DOI:** 10.1038/s41598-023-44351-9

**Published:** 2023-10-13

**Authors:** Amir Mojarrad, Reza E. Sabzi, Masoud Faraji

**Affiliations:** 1 Department of Analytical Chemistry, Chemistry Faculty, Urmia University, Urmia, Iran; 2 Electrochemistry Research Laboratory, Department of Physical Chemistry, Chemistry Faculty, Urmia University, Urmia, Iran

**Keywords:** Biocatalysis, Electrocatalysis

## Abstract

This paper introduces a unique and simple method for fabricating of inexpensive electrocatalysts for use in direct methanol fuel cells. The leached Fe_1_–Pd_1_ NFs/graphite (leached Fe_1_–Pd_1_/graphite) disk electrode was successfully obtained via uniform dispersion of Zn powder into the matrix of commercial graphite powder (98%), pressing under optimized pressure followed by the treatment in H_2_SO_4_ solution containing Fe^+2^ and Pd^+2^ cations, leading to the partial leaching out of Zn from graphite matrix, as well as partial electroless substitution of Fe–Pd nanoflakes with Zn metal. Based on the morphology studies, binary Fe–Pd nanoflakes with a large surface area uniformly dispersed on the leached graphite disk. The leached Fe–Pd/G disk showed the exceptional electrocatalytic activity toward methanol and formic acid oxidation without electrocatalyst poisoning being observed, in contrast to the leached Pd/graphite and leached Fe/graphite disks. This is due to the high surface area, and synergistic effect of Pd and Fe. The findings of this work may be used for the mass manufacture of graphite-based disks for commercial fuel cell applications using available graphite powders. The linear range of washed Fe_1_–Pd_1_/G electrocatalyst for measuring methanol was about 0.1–1.3 M, and its detection limit was calculated at about 0.03 M. Furthermore, the linear range of the nanocatalyst for measuring formic acid was about 0.02–0.1 M, and its detection limit was calculated at about 0.006 M.

## Introduction

Numerous studies looking at the electrocatalytic characteristics of platinum group metals and their alloys on carbon substrates have been carried out recently. Investigating the electrocatalytic characteristics of this type of nanocatalysts in fuel cells has been one of the most significant applications^1,2^. However, commercialization has not been suitable for these catalysts in terms of their high cost. Furthermore, these catalysts are easily poisoned by impurities, and anodic reaction products. Therefore, the use of new platinum-free catalysts, especially using the metal alloys, is of particular importance, both in terms of cost reduction and resistance to poisoning caused by intermediates^3–5^. It was tried to improve the electrocatalytic properties of fuel cell and solve its limitations. Shape and composition engineering of palladium-based materials at the nanoscale improves the catalytic activity and stability of direct alcohol fuel cells^6,7^. There are many methods used to prepare palladium-based electrocatalysts that are effective for the oxidation of alcohols, and methanol. Methods, such as synthesis of palladium colloids, sol–gel, microemulsion, electrodeposition of films, nanowires and palladium nanoparticles are considered such methods^8–11^. Compared to platinum, palladium is an attractive alternative electrocatalyst, so researchers have developed electrocatalysts based on the palladium metal for the oxidation of alcohols in alkaline environments. Instead of platinum, cheap metal alloys such as iron and palladium were used in fuel cell anodes. Pure formic acid is a colorless, toxic, corrosive and completely soluble in water, ether and alcohol. This material is used as a chemical intermediate, solvent and disinfectant. This substance is mostly used as a preservative and antibacterial in animal feed^12,13^. Formic acid is only carboxylic acid that can participate in addition reactions with alkenes. Numerous studies on the oxidation of formic acid have been undertaken recently, demonstrating its critical relevance^14,15^. Although the nanostructure is one of important features of catalyst, it is not a sufficient feature for the high activity or stability of catalyst. Other important factors, such as morphology, shape, distribution of metal particles and materials used as a substrate are necessary to improve the electrocatalyst behavior in the oxidation of alcohols^16–18^. Unlike conventional catalysts, nanocatalysts can have improved properties in terms of their leached structure, and therefore high surface area. It is used as a substrate for making nanocatalysts^19^. Given their large specific surface area, low density, and cheap cost, leached bimetallic nanostructures have recently attracted a lot of attention^20–23^. Palladium-based bimetallic catalysts exhibit better catalytic activity than palladium, according to research. The cost of manufacturing the catalyst may be decreased while the catalytic activity of the electrocatalyst is increased by alloying palladium with other transition metals. Additionally, the catalyst's electrocatalytic activity is influenced by a number of variables, including its size, shape, geometric effect, synergistic impact, etc^24,25^. In addition to reducing the amount of palladium used, alloys based on palladium also exhibit greater electrocatalytic activity. To increase the activity of novel palladium-based electrocatalysts, several attempts have been made^26,27^. Combining palladium with other transition metals, such as Fe, Cu, Cd, Ni, Co, Mn, and Sn, is one tactic that might be used^28–30^. Additionally, compared to commercial Pd/C catalysts, PdCu, PdAg, PdAu, and PdNi bimetallic catalysts shown much greater catalytic activity and stability in the electrooxidation of formic acid and methanol^31–34^. The efficacy and stability of electrocatalysts for the methanol oxidation process are enhanced by iron, a low-cost alloy metal with great strength. Fe–Pd alloys with various nanostructures, including spheres, rods, core-shells, leaves, and ultra-thin wires, have drawn a lot of attention among alloys based on palladium because of their excellent catalytic performance in reactions involving the oxidation of ethanol, oxygen reduction, and dechlorination^35,36^. According to excellent electrocatalytic performance of Fe–Pd composite towards electro-oxidation of various materials reported in pervious literatures, in this work, deposition of Fe–Pd nanoflakes onto porous graphite disks for both methanol and formic acid electrooxidation was carried out^36,37^. It is proved that the porous substrates with high surface area for deposition of electroactive material for electrocatalytic oxidation/reduction was important, where porous substrates can significantly increase active sites^38,39^. In this work, porous graphite disks for deposition of electrocatalyst material was fabricated via uniform dispersion of Zn powder into the matrix of commercial graphite powder (98%), pressing under optimized pressure followed by the leaching of Zn metal in Sulfuric acid solution as well as H_2_ bubbling^40^. In this research, the linear range of washed Fe_1_–Pd_1_/G electrocatalyst for measuring methanol is about 0.1–1.3 M, and its detection limit is calculated at about 0.03 M. Also the linear range of the nanocatalyst for measuring formic acid is about 0.02–0.1 M, and its detection limit is calculated at about 0.006 M.

## Experimental sections

### Materials and methods

Powdered graphite (98%), Powdered Zn, Iron (II) chloride (FeCl_2_), Iron(II, III) oxide (Fe_3_O_4_),, Formic acid (HCOOH), sulfuric acid (H_2_SO_4_ 98%), Sodium hydroxide (NaOH), hydrochloric acid (HCl 37%), Oleic acid (C_17_H_33_COOH), Sodium Citrate(Na_3_C_6_H_5_O_7_), ethanol (C_2_H_5_OH 99.7%), Palladium chloride (PdCl_2_) and methanol (CH_3_OH 99%) were purchased from Merck (Darmstadt, Germany). The production of graphite disks included the use of the CARVER type 3925 hydraulic press machine, which was manufactured in the United States. In addition, electrochemical measurements were conducted using an Iranian SAMA 500 electroanalytical instrument manufactured in Isfahan. Throughout the whole of the investigation and for all solutions, a solvent consisting of double-distilled water, sourced from the DD Water Company in Iran, was used. In all experiments, a three-electrode system, including a leached graphite disk working electrode with an average cross-sectional area of 0.09 cm^2^, a platinum auxiliary electrode, and a reference electrode saturated with Ag/AgCl (Azar Electrode Company, Iran) was used.

### Fabrication of the leached and non-leached graphite disks

To prepare graphite disks, 0.7 g of graphite powder was mixed with 0.3 g of Zn powder, and it was ground in a porcelain mortar for 15 min to make the mixture uniform. Using a press machine to apply 18,000 Pascal of pressure to the mixed powder, a graphite disk with a one-centimeter diameter and 0.3-mm height was produced. The graphite disk was then heated in an electric furnace for 12 h at 150 degrees Celsius. Two hours of soaking in a 1 M sulfuric acid solution increased the electrode's surface area by dissolving some Zn on the disk's surface, making it porous. The manufactured graphite disks were rubbed with sandpaper to conduct electrochemical experiments and measure the cross-sectional area of electrodes encased in plastic coverings. The cross-sectional area of the electrodes was determined to be 0.09 cm^2^. The non-leached graphite disk was made in the absence of Zn, as well as the leached graphite disk based on the above method. In the next step, nanoparticles containing Fe and Pd were easily deposited on the porous graphite disk surface via the electroless method.

### Fabrication of the leached(porous) Fe–Pd/G electrocatalyst

For preparing leached Fe–Pd/graphite (porous Fe–Pd/G) electrocatalyst, a mixture of 0.02 M of Fe_3_O_4_ and 0.005 M of PdCl_2_ was prepared in HCl solution under sonication for 30 min to obtain the source of uniform Pd^+2^ and Fe^+2^ solution. Then, to deposit Fe and Pd nanoparticles on the surface of graphite disks, the prepared previous leached (porous) graphite disks were placed inside Pd^+2^ and Fe^+2^ solution for 1.5 h. The reactions performed in the electroless process are presented in below, where Pd^+2^ and Fe^+2^ can be reduced by extra Zn available in the surface of porous graphite disk.

$$ {\text{Zn }} + {\text{ Pd}}^{{{2} + }} \left( {{\text{aq}}} \right) \leftrightarrow {\text{ Zn}}^{{{2} + }} + {\text{ Pd }}\left( {\text{s}} \right),\, {\mathbf{\Delta E}}^{{\mathbf{0}}}_{{{\text{Reaction}}}} = \, + {1}.{747,} $$

$$ {\text{Zn }} + {\text{ Fe}}^{{{2} + }} \left( {{\text{aq}}} \right)\, \leftrightarrow \,{\text{Zn}}^{{{2} + }} + {\text{ Fe }}\left( {\text{s}} \right) ,\, {\mathbf{\Delta E}}^{{\mathbf{0}}}_{{{\text{Reaction}}}} = \, + 0.{32}{\text{.}} $$

Finally, the obtained electrodes were washed with distilled water to remove the remains of chemical substances stuck to the surface of electrode.

## Results and discussion

### X-ray diffraction (XRD) and X-ray photoelectron spectroscopy (XPS) analysis

**Figure 1:**
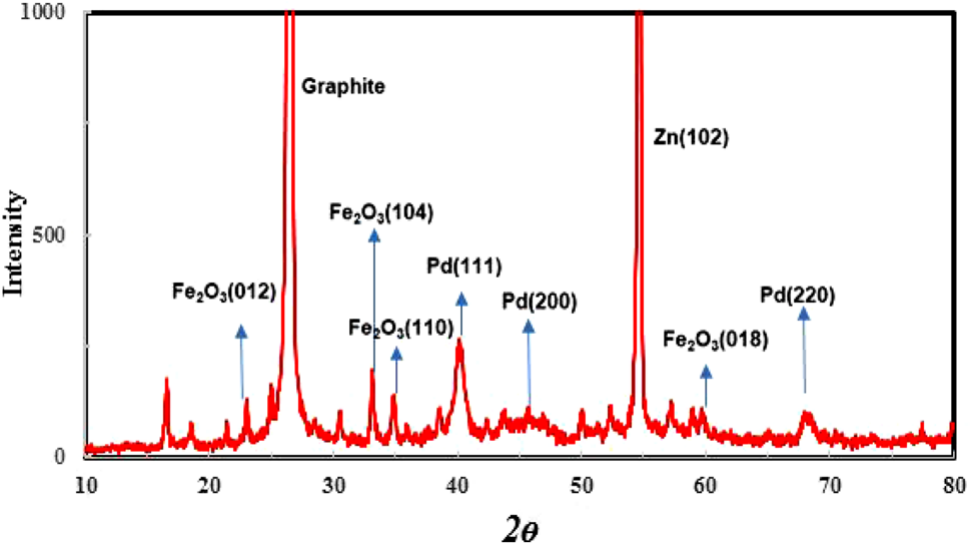
X-ray diffraction spectrum of the leached Fe_1_–Pd_1_/G electrocatalyst Loaded on a graphite substrate.

**Figure 2:**
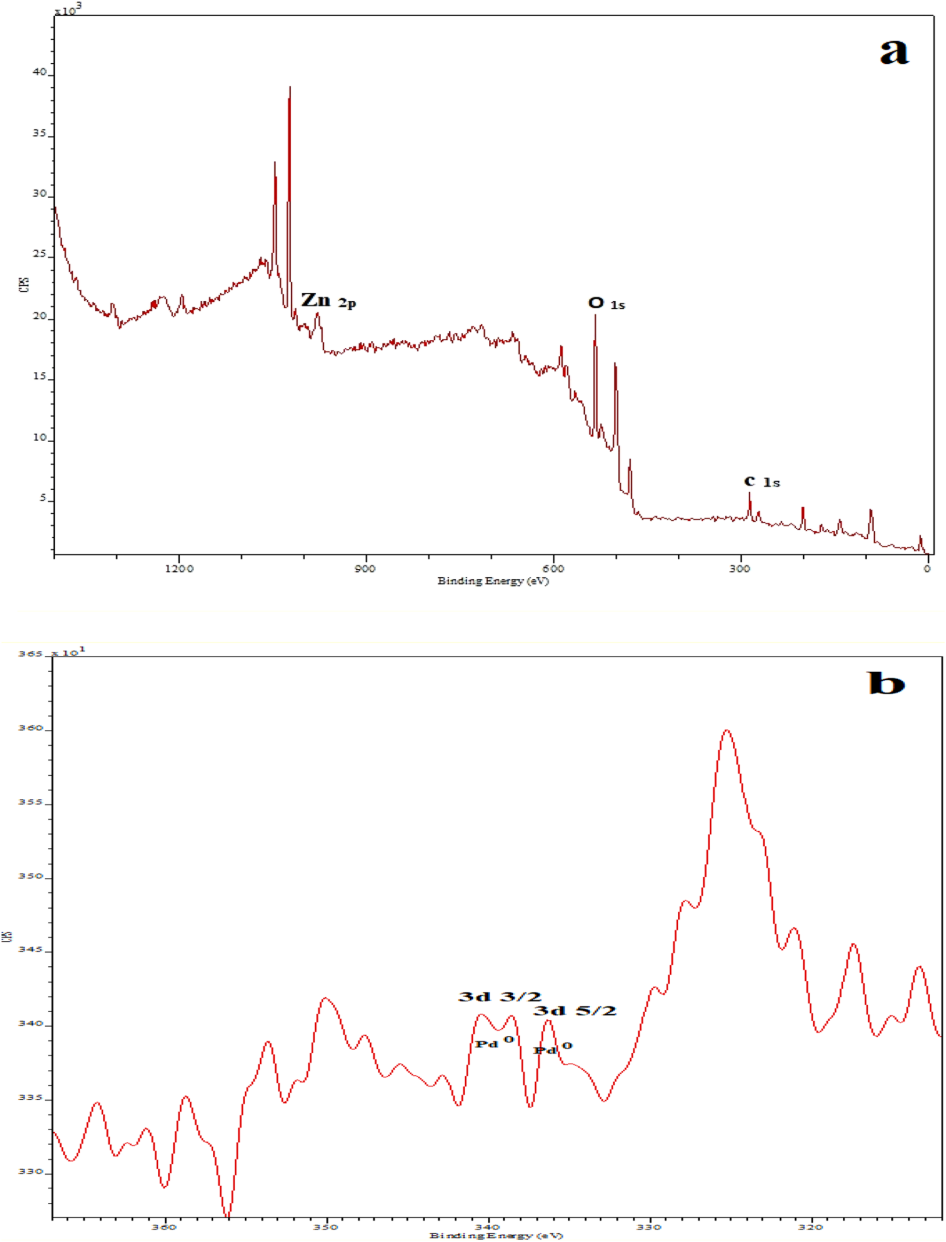

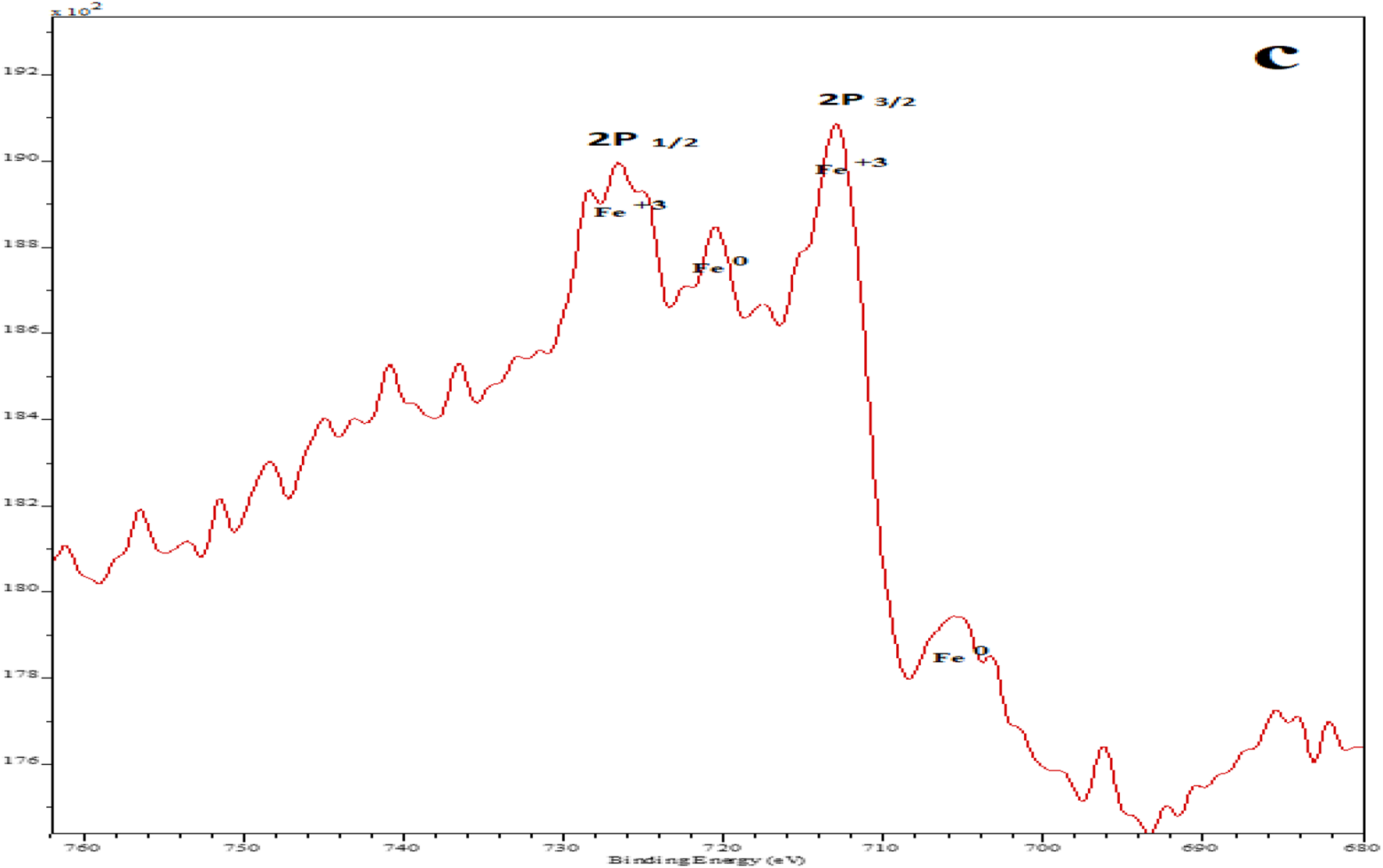
XPS measurement of the Fe–Pd /G electrocatalyst.

XRD analysis was repeated. Figure 1 shows X-ray diffraction pattern of leached Fe–Pd /G electrocatalyst, which confirms the presence of Fe_2_O_3_-Pd nanoflakes in fabricated electrocatalyst. The peaks observed at angles 26° and 55° are related to the reflection of graphite, and Zn, respectively^41,42^. Observed weak peaks at angles 41°, 46° and 68° can be attributed to crystalline structure of Pd (111), Pd (200) and Pd (220), respectively^43^. Fe (200) contributes to the peak at angle 23. Different crystalline structures of Fe_2_O_3_ are responsible for the diffraction peaks at 23, 33, 34.5, and 59, respectively^44^. The XRD analysis may have detected additional peaks due to the usage of other raw materials to produce commercial graphite. X-ray photoelectron spectroscopy (XPS) was used to get a better read on the Fe–Pd/G electrocatalyst's chemical make-up. As can be seen from Fig. 2, A doublet peaks centered at 335.1 and 340.3 eV are assigned to Pd^0^^45,46^. Four significant peaks were observed in Fe–Pd/G electrocatalyst at the binding energies of 710.7, 725.2, 707, and 720 eV, which can be ascribed to Fe^+3^ (2p3/2), Fe^+3^ (2p1/2), Fe^0^(2p3/2), and Fe^0^(2p3/2), respectively^47^. The presence of Fe(III)-O species can be attributed to Fe_2_O_3_, FeOOH, and Fe_3_O_4_, showing freshly prepared Fe(0) has an envelope of iron oxides^47^.

### Investigating the morphology of the synthesized electrocatalysts

**Figure 3:**
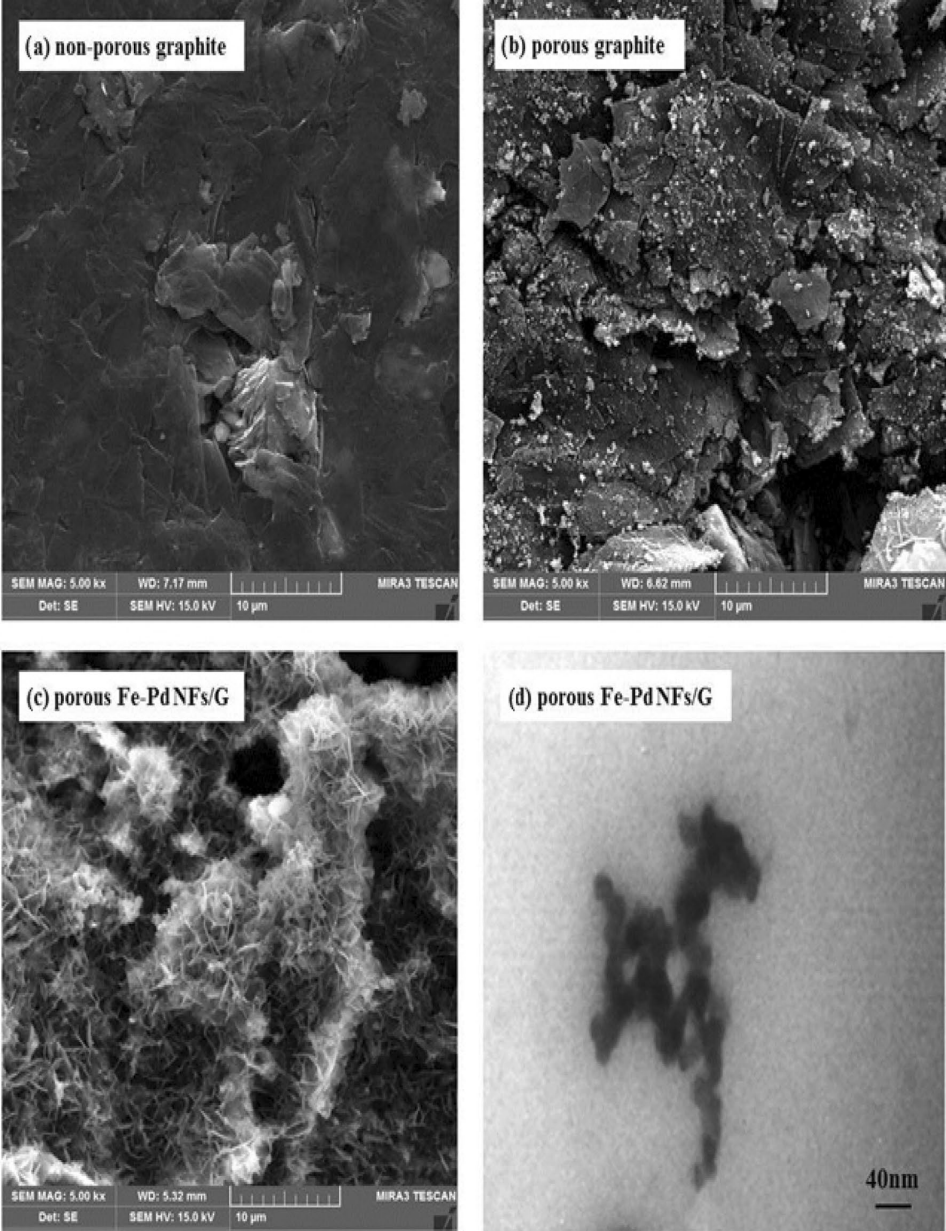

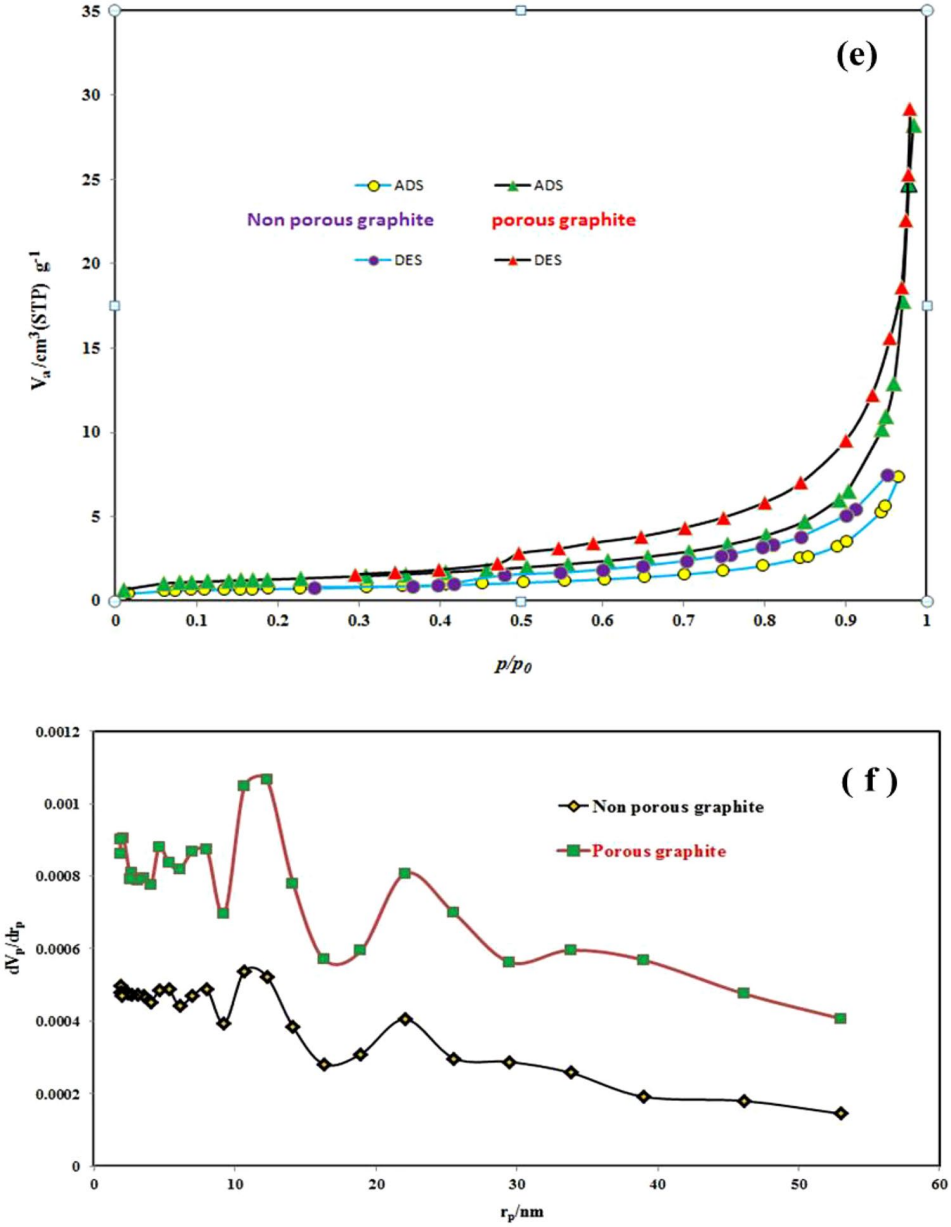
SEM images of electrocatalysts (**a**) non- leached graphite, (**b**) The leached graphite, (**c**) The leached Fe_1_–Pd_1_/G (**d**) TEM image of leached Fe_1_–Pd_1_/G electrocatalyst, (**e**) BET N_2_ adsorption–desorption isotherm (**f**) BJH pore size distribution.

Scanning electron microscopy **(**SEM) and Transmission electron microscopy (TEM) methods were used to examine the surface morphology of produced electrocatalysts, as shown in Fig. 3 SEM pictures acquired from electrocatalysts (a) non-leached graphite (b) the leached graphite (c) The non-leached graphite electrode was shown as Fe_1_–Pd_1_/G. Figure 3a There is no porosity, but the leached graphite disk in Fig. 3b clearly shows the porosity due to the placement of graphite disks in sulfuric acid and the Zn dissolving on the surface of the graphite disks, which increases the porosity. For the oxidation process, methanol was added to the solution containing the electrode. In Fig. 3c, the compact, and non-leached structure of the leached Fe_1_–Pd_1_ /G disk can be seen, completely covered by the Fe–Pd layer. SEM findings show that a highly leached catalytic surface for use in methanol electrooxidation was produced due to the deposition, and substitution of iron with zinc in the base medium. TEM picture of the surface of the graphite electrode treated with Fe–Pd nanoflakes is shown in Fig. 3d. Based on the TEM data, the fabricated nanocatalyst has an average particle size of 40 nm, resulting in increased active surface area. The results of the the Brunauer–Emmett–Teller (BET) study showed a larger specific surface area. BET isotherm and Barrett, Joyner, and Halenda (BJH) (Barrett, Joyner, and Halenda) analyses of the pore size distribution of a leached and non-leached graphite disk are shown in Fig. 3e,f. The surface area, volume, and pore diameter of the leached graphite were greatly enhanced after its treatment in sulfuric acid, as shown in Fig. 3e,f and (Table 1)^48^.

**Table 1:** BET isotherm and BJH pore size distribution analysis of the leached and non- leached graphite disk.

Sample	BET surface area (m^2^/g)	Average pore diameter (nm)	BJH pore volume (cm^3^/g)
Non-the leached graphite	2.14	15.226	0.013
The leached graphite	6.05	38.212	0.046

### Investigation of electrocatalyst hydrophilic properties

**Figure 4:**
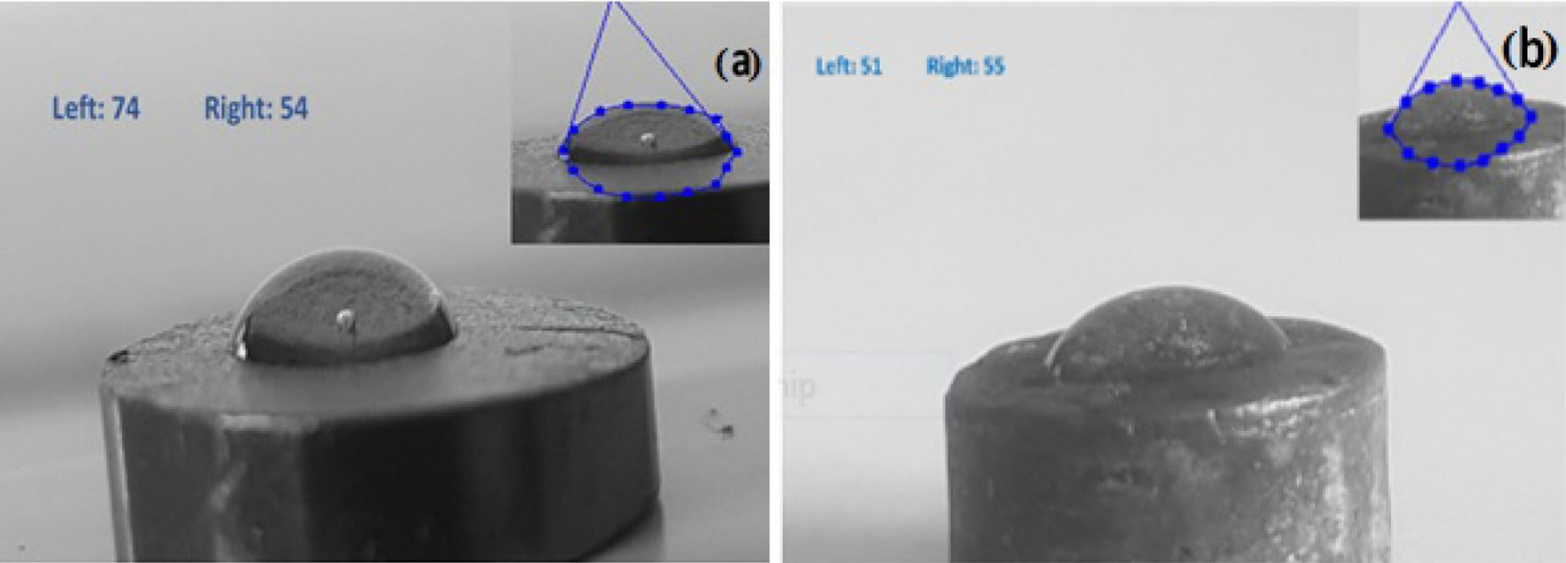
Drop test images related to electrocatalysts (**a**) non- leached graphite and (**b**) The leached graphite.

Porosity is created, and the electrode's surface area increases, boosting the hydrophilic characteristic. Parts (a) and (b) of Fig. 4 show the computed contact angles of distilled water with the leached and non-leached graphite disk electrode surfaces, respectively. Due to the difference of 11 degrees in the average contact angles between the two electrodes, 64 degrees for the non-leached graphite electrode and 53 degrees for the leached graphite electrode, it is discovered that the modified leached graphite electrode has hydrophilic properties and surface area. Decreasing the contact angle of electrode can significantly improve the electrocatalytic activity of the electrode due to increase the wettability for electrooxidation of methanol molecules dissolved in water^48^.

### Energy dispersive X-ray (EDX) elemental analysis and mapping

**Figure 5:**
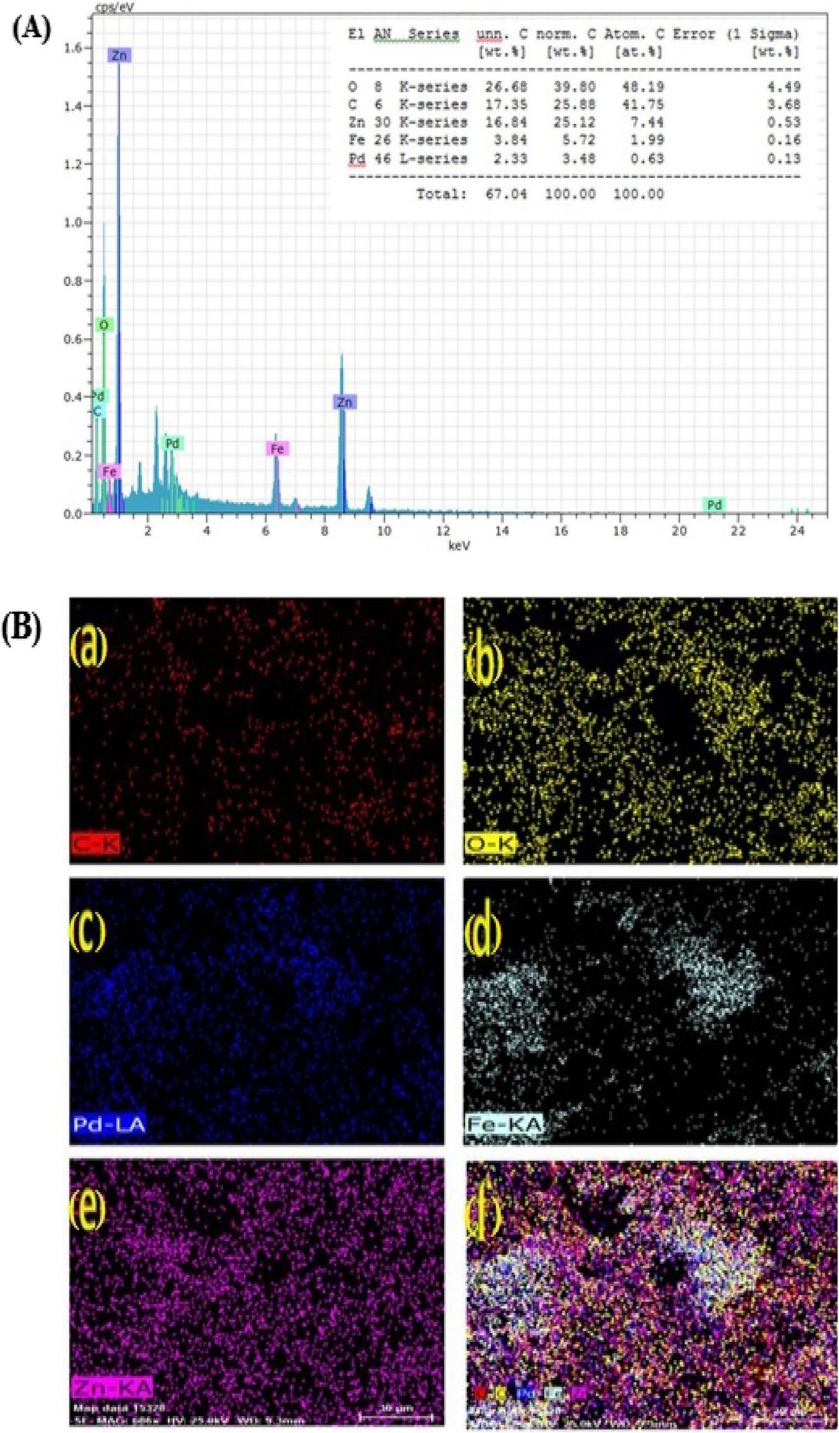
(**A**) EDX analysis of the leached Fe_1_–Pd_1_/G electrocatalyst loaded on the graphite substrate (**B**) elemental mapping (a) C, (b) O, (c) Pd, (d) Fe, (e) Zn and (f) C/O/Pd/Fe/Zn.

Figure 5A shows the results of EDX elemental analysis performed by the leached Fe_1_–Pd_1_ /G nanocatalyst. As can be seen in Fig. 5A, the above nanocatalyst is composed of the elements zinc, iron and palladium, carbon and oxygen. The percentage of elements used in the leached Fe_1_–Pd_1_/G electrocatalysts is 1.99% iron, 7.44% zinc, 0.63% Pd, 48.19% oxygen and 41.75% carbon. Based on EDX results, using Pd (0.63%) less than Fe (1.99%) is approximately (1:3), reducing the cost of making the leached Fe_1_–Pd_1_/G electrocatalysts, and this cost reduction, along with the simplicity of making electrocatalysts from the main advantages of making the above catalyst are for the oxidation of methanol. Figure 5B shows the elemental mapping of (a) C, (b) O, (c) Pd, (d) Fe, (e) Zn and (f) C/O/Pd/Fe/Zn from (a to f), respectively. Based on the figure, the elements Pd and Fe are well observed on the graphite substrate dispersed, which increases the catalytic activity of leached Fe_1_–Pd_1_/G electrocatalyst.

### Optimization of the leached Fe–Pd/G electrocatalyst for methanol electrooxidation

**Figure 6:**
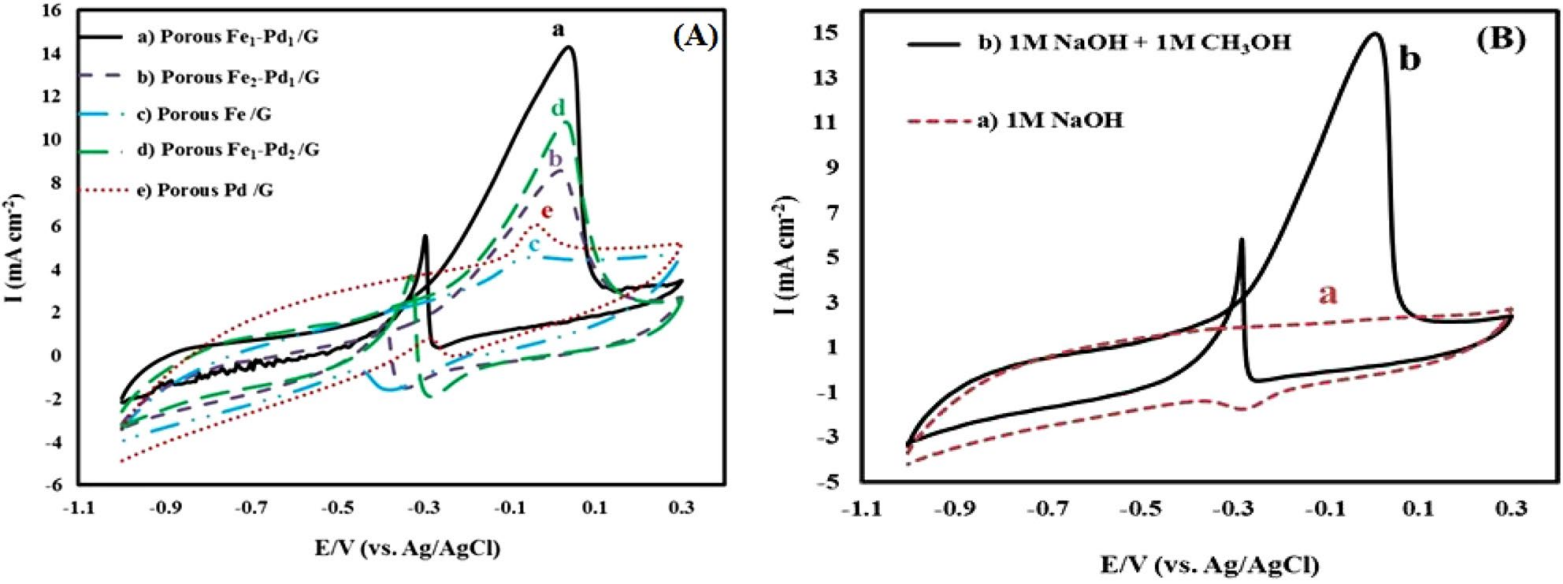
(**A**) Cyclic voltammograms of electrocatalyst the leached Fe–Pd/G with different ratios of Fe and Pd in (1 M NaOH + 1 M methanol) with a potential scan rate of 20 mV/s. (**B**) cyclic voltammograms of the leached Fe_1_–Pd_1_/G electrocatalyst in (a) 1 M NaOH and (b) (1 M NaOH + 1 M methanol) with a potential scan rate of 20 mV/s.

Figure 6A shows the cyclic voltammograms of the leached Fe–Pd/G electrocatalysts with various Fe and Pd ratios. By comparing cyclic voltammograms of electrocatalysts (a) the leached Fe_1_–Pd_1_/G, (b) The leached Fe_2_–Pd_1_/G, (c) The leached Fe/G, (d) The leached Fe_1_–Pd_2_/G and (e) The leached Pd/G in 1M NaOH current density recorded by above catalysts from (a to e) are 14.27 mA, 10.77 mA, 8.52 mA, 6.02 mA and 4.54 mA, respectively. Higher current density of methanol oxidation by electrocatalyst is observed. The leached Fe_1_–Pd_1_/G is in terms of its non-leached structure and the synergistic effect of Pd and iron, which indicates the higher catalytic activity of leached Fe_1_–Pd_1_/G electrocatalysts than other catalysts. Furthermore, the leached Pd/G is a weak catalyst for methanol oxidation compared to the leached Fe_1_–Pd_1_/G. Our major goal in developing Fe–Pd alloy electrocatalysts is to boost the modified electrode's sensitivity for methanol detection thanks to the iron alloy's synergistic effects. Increases Pd resistance to poisoning by catalytic surfaces^49^. In fact, the combination of iron and palladium, known as the electrode catalytic activity, performs a catalytic function to enhance the electrocatalyst sensitivity for methanol detection^50^. In other words, at the surface of the graphite electrode modified with Fe–Pd alloy nanoparticles, the electron transfers rate rises and the anode current increases for the oxidation of methanol. Compared to the electrocatalysts discussed above, the leached Fe_1_–Pd_1_/G catalysts exhibit a higher current density, which typically indicates an increase in the catalyst's active surface area for methanol oxidation. This increases the catalytic activity of the leached Fe_1_–Pd_1_/G and the efficiency of direct methanolic alkaline fuel cells. Based on Fig. 6A, using the ratios of (1:1) palladium to iron for the oxidation of methanol shows a higher electrocatalytic activity than other electrocatalysts, so to make a catalyst for the oxidation of methanol, the ratio of (1:1) palladium to Iron was selected. The comparison of the sensitivity of the leached electrode made for the oxidation of methanol with the electrodes prepared by previous researchers is given in (Table 2), which shows the higher sensitivity of this electrode compared to other electrodes. Then, the oxidation peak of formic acid appeared at low potential, which indicates the higher efficiency of this electrode. Figure 6B shows the cyclic voltamograms of methanol oxidation by the electrocatalyst leached Fe_1_–Pd_1_/G in (a) 1 M NaOH and (b) (1 M NaOH + 1 M of methanol). In curve 6(a), there is no anode peak in the absence of methanol, according to Fig. 6B. In curve 6(b), methanol anode peaks (forward and reverse) due to methanol oxidation, at 0.18 potentials, respectively, are there, but they are not noticed, and only a Pd reduction peak at the (− 0.1) volts potential is visible. Moreover, 0.45V, with a broad-shoulder anode peak in the positive scan in the potential range from − 0.4 to 0.4V, is related to an increase of 1 M of methanol in the electrolyte. reverse peak scan mainly in terms of the removal of carbon species in the forward scan not completely oxidized is related to the oxidation of newly adsorbed chemical species^51^.

**Table 2:** Comparative performance of this as-prepared sensor and some others for the determination of methanol.

Catalyst	Scan rate (mVs^−1^)	Sensitivity (µA mM^−1^ cm^−2^)	Potential peak V vs (Ag/AgCl)	Ref
Pt/BGA	20	38.56	0.7	^52^
Pt/NGA	20	18.84	0.65	^52^
Pt-Fe/rGO	20	4.85	− 0.14	^53^
Pd/CNT-OH	50	3.55	− 0.18	^54^
FeCo@Fe@Pd/CNT-OH	50	20.55	− 0.15	^54^
NGN/Pt	50	8.16	− 0.06	^55^
NGN/Pd	50	1.75	− 0.16	^55^
NGN/PdPt	50	8.15	− 0.15	^55^
The leached Pd /G	20	3.89	− 0.03	This work
The leached Fe_1_–Pd_1_ /G	20	13.65	0.06	This work

### Behavior of the leached Fe_1_–Pd_1_/G electrocatalyst for oxidation of methanol

**Figure 7:**
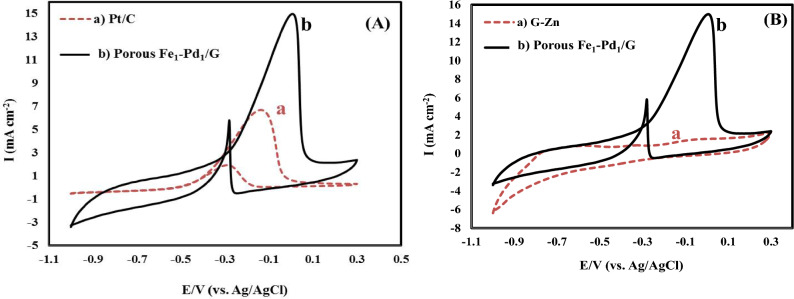
(**A**) Cyclic voltammograms by Fe_1_–Pd_1_/G, Pt/C electrocatalysts of methanol oxidation in (1 M NaOH + 1 M methanol). (**B**) cyclic voltammograms by Fe_1_–Pd_1_/G, G/Zn electrocatalysts for methanol oxidation in (a) 1 M NaOH and (b) (1 M NaOH + 1 M methanol) with a potential scan rate of 20 mV/s.

**Figure 8:**
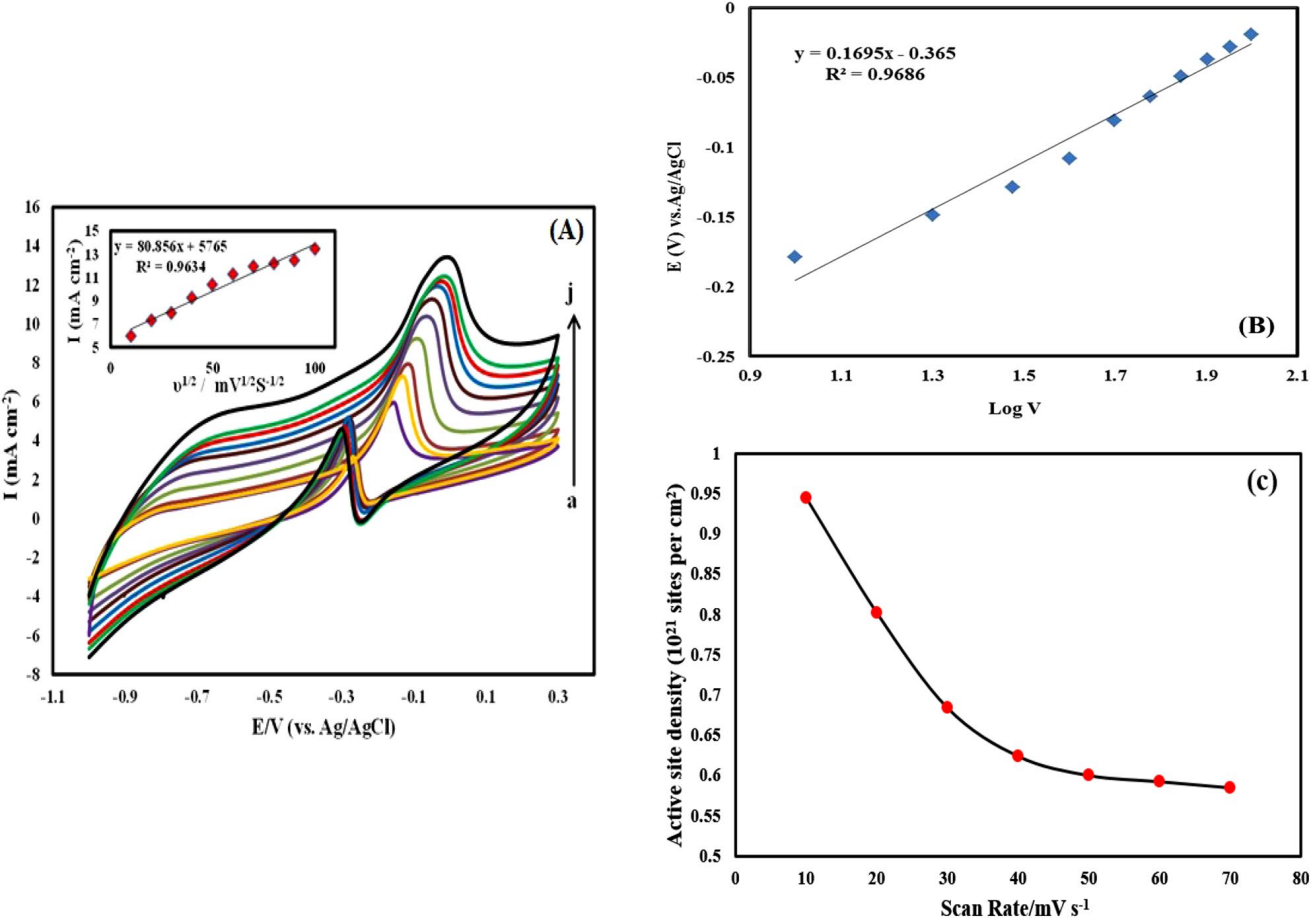
(**A**) Cyclic voltammograms of methanol oxidation by leached Fe_1_–Pd_1_/G electrocatalyst at different scan rates of 10, 20, 30, 40, 50, 60, 70, 80, 90, 100 mV/s respectively from (a to j) in (1 M NaOH + 0.3 M methanol). (**B**) Peak potential variation graph in terms of the logarithm of potential scan rate related to cyclic voltammograms for leached Fe_1_–Pd_1_/G electrode. (**C**) The estimated active site density per unit cm^2^ of catalyst at different scan rates for leached Fe_1_–Pd_1_/G electrocatalyst electrode.

Comparing cyclic voltammograms of Fe_1_–Pd_1_/G, Pt/C electrocatalysts for the methanol oxidation is shown in Fig. 7A. As can be observed, Fe_1_–Pd_1_/G electrocatalyst compared to Pt/C electrocatalyst for methanol oxidation showed a higher current intensity, which indicates better performance of Fe_1_–Pd_1_/G electrocatalyst compared to Pt/C electrocatalyst for methanol oxidation. Furthermore, cyclic voltammograms of Fe_1_–Pd_1_/G, G/Zn electrocatalysts for methanol oxidation were studied.as can be seen in Fig. 7B, for non-porous G/Zn electrocatalyst, which lacks Fe–Pd. No peak for methanol oxidation was observedwhich indicates the lack of effect of Zn on methanol oxidation. Cyclic voltammograms of the leached Fe_1_–Pd_1_/G nanocatalyst oxidizing methanol at various scan speeds are shown in Fig. 8A. As can be observed, by raising the scan rate from 10 to 100 mV/s, the methanol oxidation-related Faraday current and non-Faradi current have both grown, which has caused the anodic peaks to become broader. The peak potential of methanol oxidation and the current density rise by increasing scanning speed in Fig. 8A. Additionally, because of excess kinetic voltage, the peak of the methanol oxidation potential changes to greater positive potentials as the scan rate increases^56^. The left-hand corner of Fig. 8A displays the curve of variations in the peak anode current intensity with the root of the scan rate. This figure illustrates the linear connection between the anode current density and the root of scan rate, with a correlation coefficient of 0.9634, showing the diffusion-controlled regulation of electrode reaction process. Figure 8B related to leached Fe_1_–Pd_1_/G nanocatalyst shows the linear changes of anodic nose potential of methanol electrooxidation due to the logarithm of potential scan rate, the slope obtained for the said line is 169/dec mV. Considering na = 1 and based on following equation, Tafel slope, and the approximate value of α are obtained as follows:

$$ {\text{b }} = {2} \times {169} = { 338} {\text{mV}}/{\text{dec}},\,{\text{n}}_{{\text{a}}} = { 1} ,\,\alpha \, = \, 0.{ 83},\,{\text{n}}_{{\text{a}}} \left( {{1} - \alpha \, } \right) \, = {59}/{388 } = 0.{17}. $$

By using the slope of the graph above and using Laviron's theory, the number of electrons involved in the oxidation of methanol can be calculated:

$$ {\text{E}}_{{\text{p}}} = {\text{ E}}^{0} + \, \left( {{2}.{3}0{\text{3 RT}}/\alpha {\text{nF}}} \right){\text{ log }}\left( {{\text{RTK}}^{0} /\alpha {\text{nF}}} \right) \, + \, \left( {{2}.{3}0{\text{3 RT}}/\alpha {\text{nF}}} \right){\text{ log v}}\,0.{169 } = { 2}.{3}0{\text{3 RT}}/\alpha {\text{nF}},\,\alpha \, = \, 0.{83},\,{\text{n}} = \, 0.{72}{\text{.}} $$

The adsorption charge measured from the voltammogram of leached Fe_1_–Pd_1_ /G electrocatalyst is used to estimate the active site density by the following equation^57^:

$${\varvec{S}}{\varvec{D}}({\varvec{a}}{\varvec{c}}{\varvec{t}}{\varvec{i}}{\varvec{v}}{\varvec{e}}\boldsymbol{ }\,{\varvec{s}}{\varvec{i}}{\varvec{t}}{\varvec{e}}\boldsymbol{ }\,{\varvec{p}}{\varvec{e}}{\varvec{r}}\boldsymbol{ }\,{\mathbf{c}\mathbf{m}}^{2})=\frac{\mathbf{I}\mathbf{n}\mathbf{t}\mathbf{e}\mathbf{g}\mathbf{r}\mathbf{a}\mathbf{t}\mathbf{e}\mathbf{d}\,\mathbf{C}\mathbf{V}\,\mathbf{a}\mathbf{r}\mathbf{e}\mathbf{a}\left(\mathbf{A}.\mathbf{V}\right)\times \mathbf{N}(\mathbf{s}\mathbf{i}\mathbf{t}\mathbf{e}\mathbf{s}\,\mathbf{p}\mathbf{e}\mathbf{r}\,\mathbf{m}\mathbf{o}\mathbf{l})}{{\varvec{n}}\times {\varvec{s}}{\varvec{c}}{\varvec{a}}{\varvec{n}}\,{\varvec{r}}{\varvec{a}}{\varvec{t}}{\varvec{e}}({\varvec{v}}.{{\varvec{s}}}^{-1})\times {\varvec{F}}({\varvec{C}}.{{\varvec{m}}{\varvec{o}}{\varvec{l}}}^{-1})\times {\varvec{s}}({\mathbf{c}\mathbf{m}}^{2})}$$

In this equation, SD is mass-specific site density, N is Avogadro number (6.023 × 10^23^ sites per mol), n is the number of electrons, F is Faraday constant (96,485 C.mol^−1^), and s is the geometric area of leached Fe_1_–Pd_1_/G electrocatalyst electrode (0.09 cm^2^). The estimated active site density per unit cm^2^ of catalyst at different scan rates for leached Fe_1_–Pd_1_ /G electrocatalyst electrode is shown in Fig. 8c.

**Figure 9:**
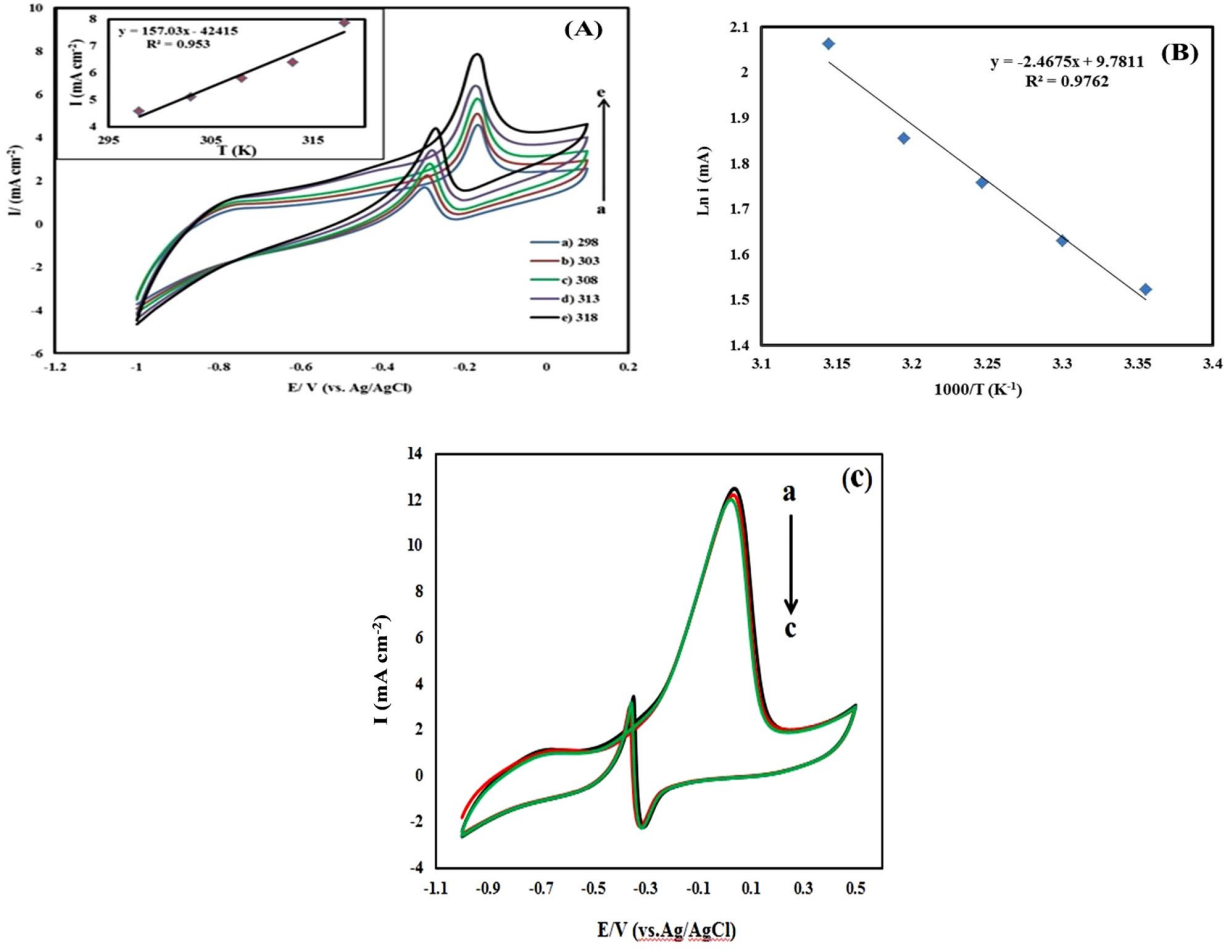
(**A**) Cyclic voltammograms of methanol oxidation by the leached Fe_1_–Pd_1_/G nanocatalyst at temperatures of 25, 30, 35, 40 and 45 °C in (1 M NaOH + 0.2 M methanol) with a potential scan rate of 20 mV/s. (**B**) Arrhenius diagram for methanol oxidation by the leached Fe_1_–Pd_1_/G nanocatalyst. (**C**) Cyclic voltammograms of methanol oxidation by the leached Fe_1_–Pd_1_/G electrocatalyst (a) 1 cycle (b) after 100 Cycle (c) after 200 cycles.

The temperature effect in the methanol oxidation process as an important factor was investigated^58,59^. The cyclic voltammetry approach was used to examine the effect of temperature on the methanol oxidation by the leached Fe–Pd/G nanocatalyst in (0.2 M methanol + 1 M NaOH) at temperatures of 25, 30, 35, 40, and 45 degrees Siliceus concurred. Figure 9A shows that when the temperature rises from 25 to 45 °C, the peak's height rises as well, indicating that the rate of methanol oxidation has risen. Moreover, it may be said that a reduction in load transfer resistance has resulted from a rise in temperature. Additionally, Fig. 9B provides an Arrhenius curve for the methanol oxidation using the leached electrode Fe–Pd/G. The activation energy value for methanol oxidation was found to be 2.46 kcal/mol using the Arrhenius diagram's slope. In addition of catalytic activity, the long-term stability of electrocatalysts is a significant criterion for evaluating their practical use^60,61^. The cyclic voltammogram of the leached Fe_1_–Pd_1_/G electrocatalyst was obtained after 200 cycles and compared to the initial cycle in order to assess the stability of the electrode and its resistance to Carbon monoxide (CO) poisoning. Compared to the first cycle, the forward peak current density in cycle 200 is decreased by about (0.3 mA) as seen in Fig. 9c, indicating excellent activity and durability of the leached Fe_1_–Pd_1_/G electrocatalyst for methanol oxidation in alkaline media.

**Figure 10:**
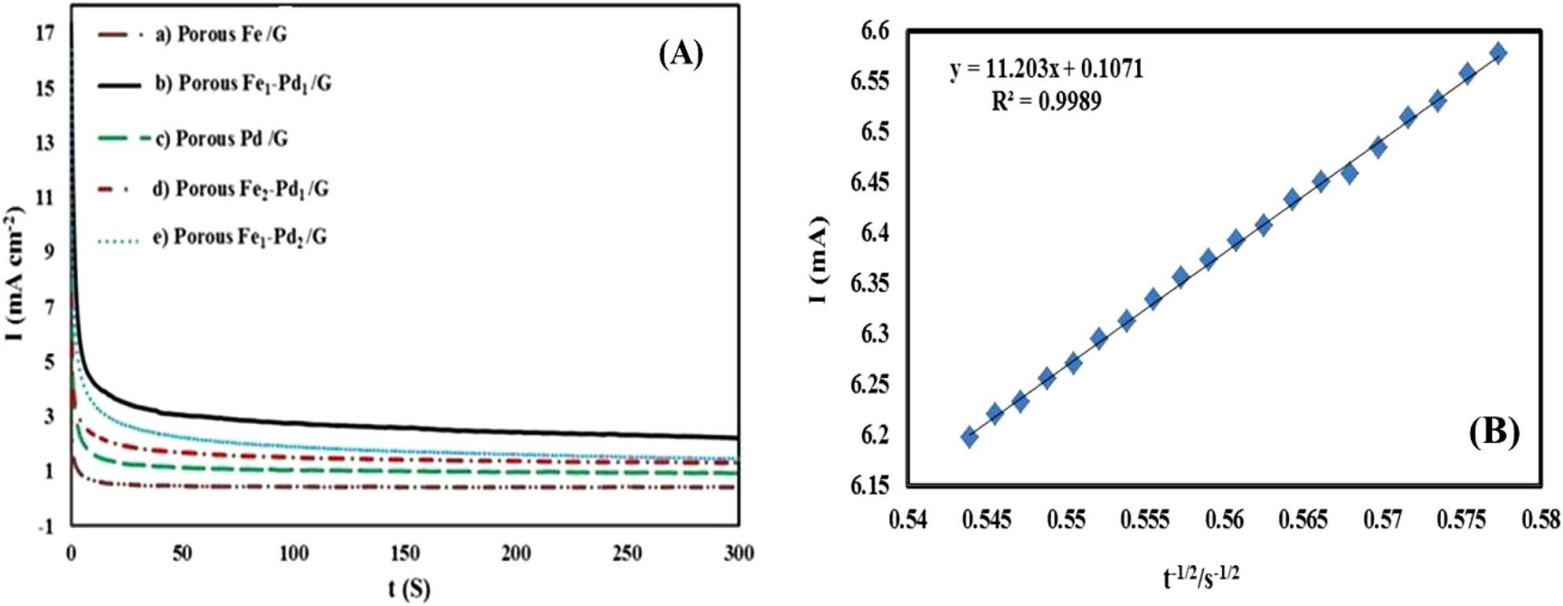
(**A**) Chronoamperograms of methanol oxidation related to electrocatalysts (a) The leached Fe/G, (b) The leached Fe_1_–Pd_1_/G, (c) The leached Pd/G, (d) The leached Fe_2_-Pd_1_/G and (e) The leached Fe_1_-Pd_2_/G in (1 M NaOH + 1 M methanol). (**B**) Plot of current versus time at 1 M methanol concentration.

The chronoamperometric (CHA) curves for all fabricated disks are shown in Fig. 10A in (1 M NaOH + 1.0 M methanol) at a constant potential of − 0.2 V for 300 s. As can be seen from the figure, all fabricated disks possess high peak current density at the beginning of the CHA test, attributing to the double layer charging process of the available huge active sites on the disk surface. As time passes in CHA test, current density was significantly reduced for all electrocatalyst disks which can be attributed to occupy and block the active sites on surface of disks due to toxic species specially CO derived from methanol electrooxidation. Finally, the current density was almost remained stable, assigning to constant diffusion of methanol species from bulk to surface disk. As can be seen from the Fig. 10A, the leached Fe_1_–Pd_1_/G electrocatalyst-disk exhibits greater stable current density (14.23 mA cm^−2^) compared to other fabricated disks for the electrooxidation of methanol, showing its higher durability against CO poising effect^58,62^.

Figure 10B shows plot of current density against the t^1/2^ of CHA test for leached Fe_1_–Pd_1_/G electrocatalyst-disk, where diffusion coefficient can be calculated. The diffusion coefficient of methanol in aqueous medium can be calculated using the following equation, which is known as Cattrell's equation. In Cottrell's relation, I: current intensity in amperes, n: number of exchanged electrons, F: Faraday number, C: concentration of electroactive compound in t: time in seconds, D: diffusion coefficient in A: electrode surface area in is. Using the Cottrell equation and the slope of the flow diagram in terms of time, the diffusion coefficient of methanol (D) was obtained 7 × 10^−4^ cm^2^.s^−1^.

$$ {\text{I}}\, = \,{\text{nFACD}}^{{{1}/{2}}} . \, \pi^{{ - {1}/{2}}} {\text{t}}^{{ - {1}/{2}}} \, \to \,{\text{D}} = { 7} \times {1}0^{{ - {4}}} {\text{cm}}^{{2}} .{\text{s}}^{{ - {1}}} . $$

**Figure 11:**
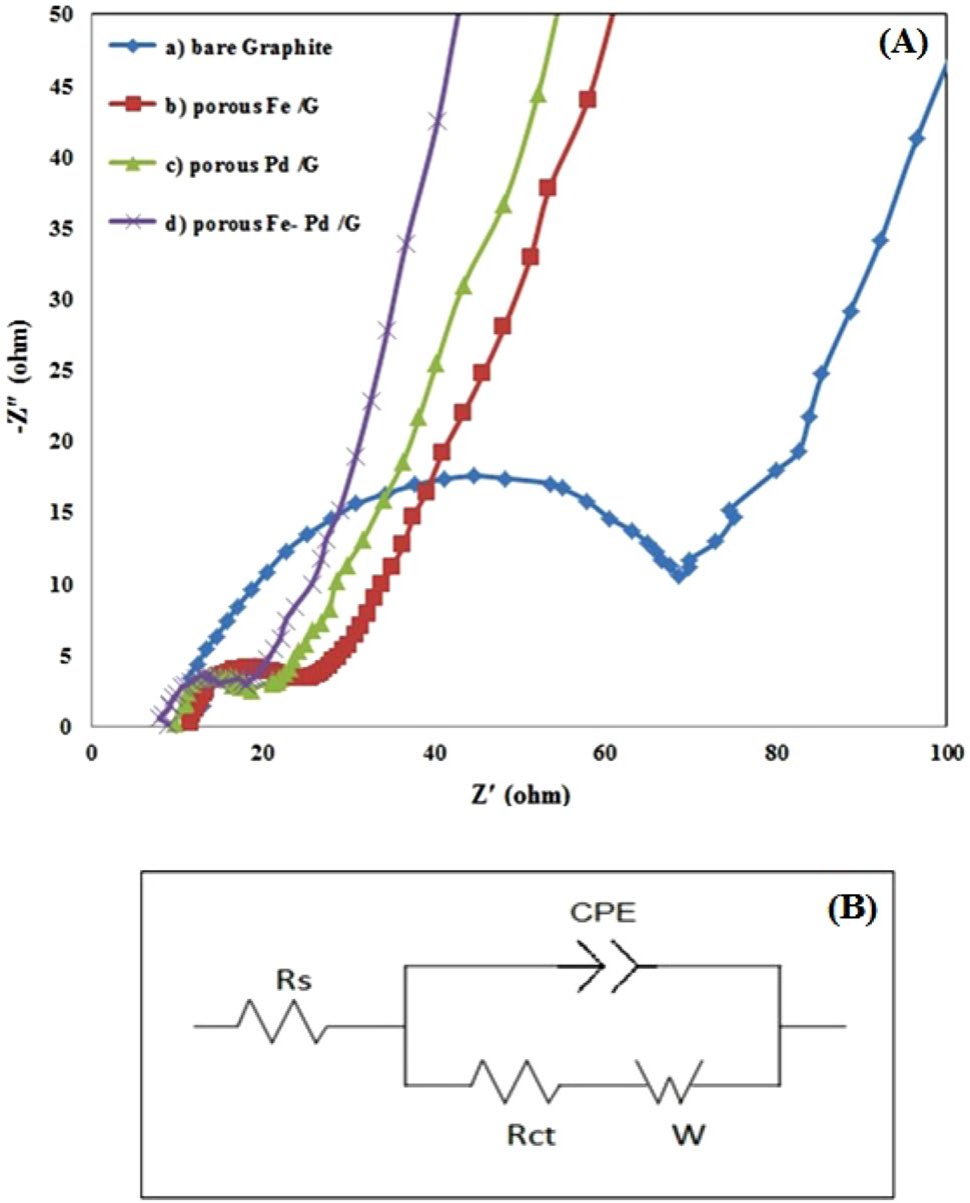
(**A**) Nyquist plots of methanol electrooxidation on (a) bare graphite, (b) The leached Fe/G, (c) The leached Pd/G and (d) The leached Fe–Pd/G electrocatalysts in 1 M NaOH + 1 M methanol at an electrode potential of − 0.20 V. (**B**) Electrical equivalent circuits.

In electrochemical systems, the kinetics of electrode reactions can be studied using electrochemical impedance spectroscopy (EIS)^63^. In 1.0 M NaOH and 1.0 M CH_3_OH at 0.20 V, typical Nyquist plane plots of the as-prepared bare graphite (a), leached Fe/G (b), leached Pd/G (c), and leached Fe_1_–Pd_1_/G (d) electrocatalysts are shown in Fig. 11A. It has been known that charge transfer resistance of electrocatalysts is determined by the semicircle's diameter^35^. The diameter of the impedance arc on the leached Fe_1_–Pd_1_/G electrocatalyst is much less than that of other electrocatalysts, as can be shown. By using Zview software and fitting the EIS data, the electrical equivalent circuit was discovered according to Fig. 11B. The constant phase element (CPE), R_s_ and R_ct_ show electrode double-layer capacitance, resistance of the solution as well as electrodes and the charge transfer resistance on the electrocatalysts, respectively^30^. Also, according to relation of i_0_ = RT/FR_ct_ (T is temperature, R is the gas constant and F is Faraday constant), exchange current density (i_0_) is calculated. The below table shows a comparison of obtained electrodes in view of exchange current density (i_0_) and charge transfer resistance (R_ct_)^64^. As can be seen from the Table 3, leached Fe_1_–Pd_1_/G possess lower R_ct_ and higher i_0_ compared to other electrodes, showing its good electrocatalytic activity compared to other electrodes which is in accordance with other electrochemical evaluations.

**Table 3:** A comparison of obtained electrodes in view of exchange current density (i_0_) and charge transfer resistance (R_ct_).

Electrodes	R_ct_	i_0_(A)
Bare graphite	61Ω	4.2 × 10^−4^
Leached Fe_2_O_3_/G	14.5 Ω	1.7 × 10^−3^
Leached Pd/G	12 Ω	2.1 × 10^−3^
Leached Fe_2_O_3_-Pd/G	10.5 Ω	2.4 × 10^−3^

### Behavior of different fabricated electrodes for oxidation of formic acid

**Figure 12:**
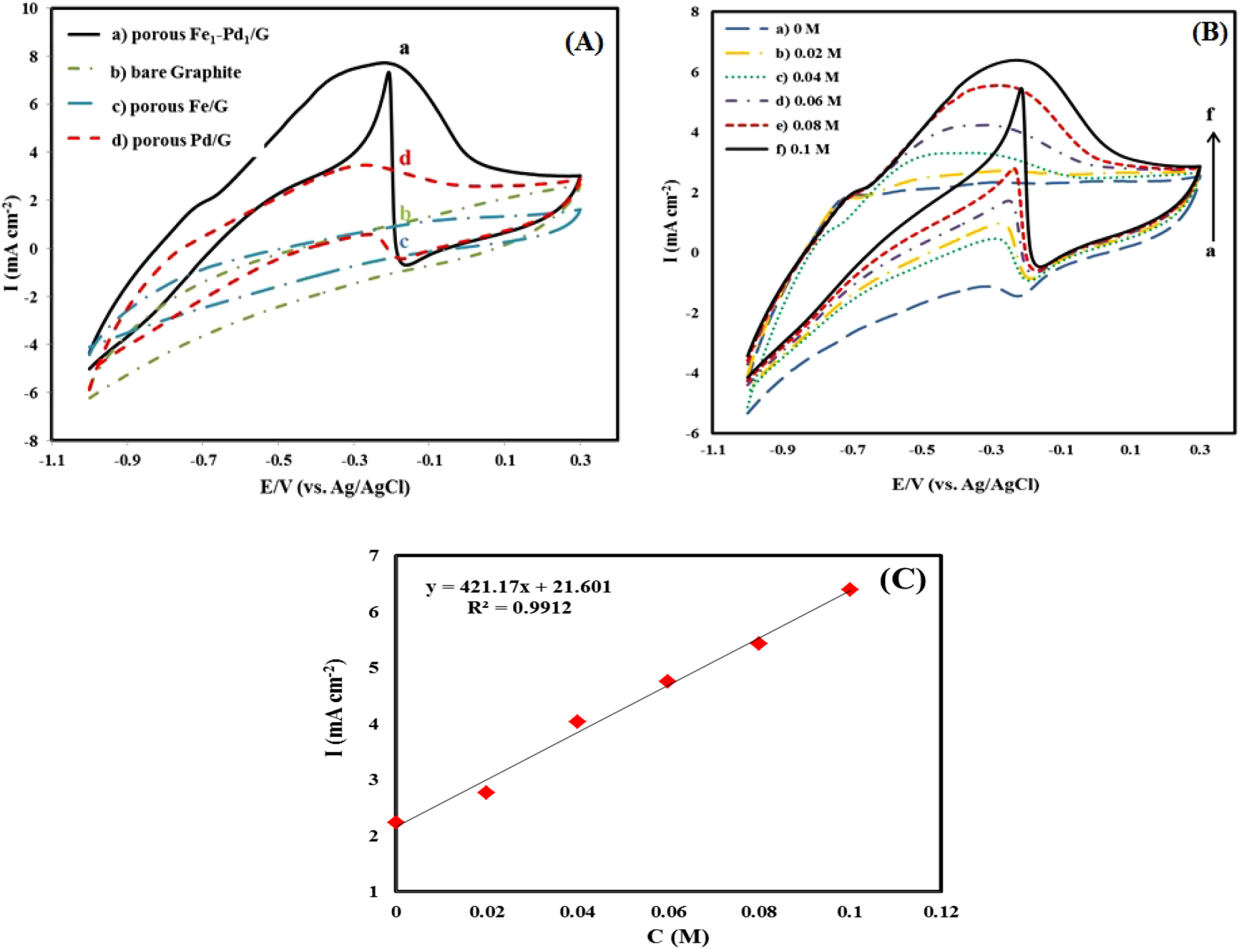
(**A**) Cyclic voltammograms of (a) The leached Fe_1_–Pd_1_/G, (b) bare graphite. (c) The leached Fe/G (d) The leached Pd/G electrocatalysts in (0.1 M H_2_SO_4_ + 0.1 M formic acid) with a potential scan rate of 20 mV/s. (**B**) Cyclic voltammograms of formic acid oxidation by the leached Fe_1_–Pd_1_/G electrocatalyst in different concentrations formic acid of 0, 0.02, 0.04, 0.06, 0.08, 0.1M respectively from (a to f) in 0.1 M H_2_SO_4_ with potential scan rate of 20 mV/s. (**C**) Plot of flow versus concentration at 0.1 M formic acid concentration.

Figure 12A compares the cyclic voltammograms of various fabricated disk-electrocatalysts in 0.1 M H_2_SO_4_ and 0.1 M formic acid at a scan rate of 20 mV/s. As observed in Fig. 12A, the leached Fe_1_–Pd_1_/G disk-electrocatalyst has maximum electrocatalytic activity towards formic acid oxidation originated from nanostructure morphology and the synergistic impact of Fe–Pd alloy. In actuality, the combination of iron and palladium serves as a catalyst to boost the modified electrode's sensitivity in oxidation of formic acid. Table 4 compares the sensitivity of the fabricated leached disk-electrocatalysts towards formic acid oxidation with other electrodes reported in previous works. As can be seen from the Table, the leached Fe_1_-Pd_1_/G disk shows more negative oxidation potential and higher current density towards formic acid oxidation compared to other previous electrodes. Figure 12B displays electrooxidation of formic acid on the leached Fe_1_–Pd_1_/G disk at various formic acid concentrations in 0.1 M H_2_SO_4_ and 20 mV/s scan rate. As formic acid concentration increases up to 0.1 M, the peak changes slightly to higher positive values, and the anode peak current density increases continuously, suggesting a large number of active nanocatalyst sites. The curve of current versus concentration at 0.1 M formic acid concentration is shown in Fig. 12C, displaying linear correlation between concentration and peak current density.

**Table 4:** Comparative performance of this as-prepared sensor and some others for the determination of formic acid.

Catalyst	Scan rate (mVs^−1^)	Sensitivity (µA mM^−1^ cm^−2^)	Potential peak V vs. (Ag/AgCl)	Ref
Pt/NGA	20	14.96	0.7	^52^
Pt/BGA	20	20.16	0.75	^52^
PtRu/C	20	4.5	0.7	^65^
PtPd/C	20	4.2	0.62	^65^
PtSn/C	20	3.5	0.63	^65^
Pd-black@CP	10	1.7	0.3	^66^
Pd-nanoarray@CP	10	10	0.6	^66^
EPPGE–MWCNT–PtFeOCPc	–	9	0.25	^67^
Pd/C	50	0.75	0.2	^68^
PdAgNi/C	50	1.75	0.25	^68^
The leached Pd Nps/G	20	30.46	− 0.27	This work
The leached Fe_1_-Pd_1_ Nps/G	20	70.72	− 0.23	This work

## Conclusion

The porous Fe–Pd NFs/G coin was prepared using facile electroless deposition of Fe–Pd nanoflakes onto porous Zn-graphite coin obtained from pressed commercial graphite and Zn powders. The porous Fe–Pd NFs/G showed enhanced electrocatalytic activity and good stability toward methanol oxidation with high anti-CO poisoning capability compared with leached Pd/G and Fe/G disks. The anodic peak for oxidation of methanol on porous Fe–Pd NFs/G electrode was almost two and three times higher than that of other electrocatalysts such as porous Pd/G electrode and porous Fe/G electrode, respectively. Compared to similar electrocatalysts, the materials used in the current disk are available, cheap and non-toxic. Therefore, the use of these disks will be very helpful in the direction of developing the use of non-fossil and clean energy systems. The results of this research can be used in industries related to new energy, in fuel cell systems, batteries and supercapacitors. Also, these nanocatalysts can be used in electrocatalytic processes to detect and measure drugs and biological species.

## Data Availability

The data produced and analyzed during the current study are available from the corresponding authors on reasonable request.
